# Measuring Causal Invariance Formally

**DOI:** 10.3390/e23060690

**Published:** 2021-05-30

**Authors:** Pierrick Bourrat

**Affiliations:** 1 Department of Philosophy, Macquarie University, Balaclava Road, North Ryde, NSW 2109, Australia; p.bourrat@gmail.com; 2 Department of Philosophy & Charles Perkins Centre, The University of Sydney, Camperdown, NSW 2006, Australia

**Keywords:** causation, invariance, causal specificity, information theory

## Abstract

Invariance is one of several dimensions of causal relationships within the interventionist account. The more invariant a relationship between two variables, the more the relationship should be considered paradigmatically causal. In this paper, I propose two formal measures to estimate invariance, illustrated by a simple example. I then discuss the notion of invariance for causal relationships between non-nominal (i.e., ordinal and quantitative) variables, for which Information theory, and hence the formalism proposed here, is not well suited. Finally, I propose how invariance could be qualified for such variables.

## 1. Introduction

The interventionist account characterizes a causal relationship between two variables *C* and *E* in the following way:

Minimal Criterion: *C* is a cause of *E* if there is at least one ideal intervention on *C* that changes the value of *E*

This version of the Criterion is borrowed from Bourrat [1]. For a much more detailed version see Woodward [2] (p. 59).

An ideal intervention on a variable is a change in the value of that variable that produces no other change on any other variable at the time of change [2] (p. 14). A more precise characterization of this criterion is found in Woodward [2] (pp. 98–99). The Minimal Criterion of causation is the core of the interventionist account.

Although the Minimal Criterion of causation can help us decide whether a given relationship is causal, it does not permit us to quantify in any way such a relationship. Yet, one might want to compare different causal relationships or even get more details on a relationship itself. Comparing causal relationships might involve, for instance, assessing whether there are a different number of possible interventions on *C* that lead to changes in *E* for these different relationships (assuming the same probability for each intervention). Capturing more details on one relationship might involve assessing whether various interventions on *C* lead to a different sort of changes in *E* across the whole range of possible interventions (assuming here again the same probability for each intervention).

To characterize more precisely a causal relationship, a number of dimensions have been proposed in addition to the Minimal Criterion, see Woodward [2], Woodward [3], Griffiths et al. [4], and Pocheville et al. [5]. Following the treatment of Woodward [2], Woodward [3], Woodward [6], two of these dimensions are invariance and specificity [3,4,5,7,8]. Woodward [3] makes a distinction between two notions of causal specificity, namely what Bourrat [1] calls “range of causal influence”, which corresponds to Woodward’s ‘INF’ (for influence) [3] (p. 305) and what Woodward calls “one cause-one effect” specificity (p. 310), hereafter ‘one-to-one specificity,’ following Bourrat [1]. One way to characterize the range of causal influence of *C* over *E* is as a measure of the number of possible interventions on *C* producing *different* changes in *E*. If different possible interventions on *C* produce the same change in *E*, the range of influence of the causal relationship between *C* and *E* will be lower than if each possible intervention leads to a different change in *E*. A classical example to illustrate the notion of range of influence comes from Woodward [3]. The dial of a radio has a higher range of influence than the on/off switch. This is so because there are many positions of the dial that correspond to many different channels. In contrast, the range of influence of the on/off switch is lower because it can only take two values and lead to two distinct outcomes: we hear something or we do not.

One-to-one specificity concerns the extent to which exactly one value of *C* corresponds to exactly one value of *E*. For instance, an enzyme might bind to only a single substrate while a different enzyme might bind to different substrates and catalyze a higher number of reactions. The binding specificity of the first enzyme, which is an example of one-to-one specificity, is higher than the specificity of the second enzyme. For more on the distinction between the two types of specificity see Bourrat [1], Woodward [3], and Lean [9].

As we shall see, range of influence has been formalized by Griffiths et al. [4] as mutual causal information (see Section 2), while Bourrat [1] has provided a formalism close to that of Griffiths et al., for one-to-one specificity. One-to-one specificity corresponds to an information-theoretic measure related to causality mutual information known as (causal) variation of information.

Moving on to invariance, Woodward defines the invariance between *C* and *E* as the extent to which this relationship “remains stable or unchanged as various other changes occur” [2] (p. 239). He also distinguishes two types of invariance [2,3,6]. A relationship is invariant following the first sense if for any ideal intervention on *C*, the causal relationship (or function) C→E remains unchanged. Most causal relationships are not invariant for all possible ideal interventions. However, the more invariant the relationship, the larger the number of ideal interventions on *C* will leave the functional relationship F(C)=E unchanged. For instance, to use an example inspired from Woodward [2], different springs made of different materials with the same stiffness in the same conditions (e.g., temperature) will satisfy Hooke’s law—which says that the force necessary to extend the spring by a given distance is proportional to that distance—with different degrees of invariance. Some springs will be able to undergo more force before breaking up, and thus the causal relationship between force and distance will be more invariant than for other, more fragile springs. Taking the radio example again with slight modifications, suppose two radios which are both susceptible to interference in presence of a cell phone. However, one radio is more susceptible to interference than the other— when a cell phone is placed in the same room as the radios, there more locations where the cell phone is placed that lead to some interference with this radio, everything else being equal. While both radios are susceptible to inference, the causal relationship between the location of the cell phone and the existence of interference with the radio is more invariant for the radio which is more susceptible to interference.

Under the second sense of invariance, a causal relationship is more invariant than a second relationship, if intervening on the value of variables in the background of the first relationship leads to less change in the properties of the relationship than changes in the properties of the second relationship. For instance, some alleles of a particular gene are expressed only in a particular environment. This is the case of a mutation causing a suite of reactions (e.g., fever, muscle rigidity) known as “malignant hyperthermia” *only* during a general anesthesia [10]. Other genes are expressed in nearly all viable environments. This the case of genetic abnormalities on the fourth chromosome leading to Huntington’s Disease, which occurs in 100% of the individuals with these abnormalities [10]. The causal relationship between genotype and phenotype in more invariant in the case of Huntington’s Disease than of malignant hyperthermia. Here again, no causal relationship is invariant for all possible interventions in the background of the relationship under this second sense. Yet, the higher the invariance of the relationship, the larger the number of interventions on the background variables, which can be symbolized as *B*, will keep the function F(C)=E unchanged. Following Pocheville et al. [5], I will call the first kind of invariance “invariance” and the second kind “stability.”

Woodward’s framework can be deployed in many different contexts. For instance, it has been applied in the context of genetic causation (e.g., [1,5,8,11,12]), developmental biology (e.g., [13,14]), heritability (e.g., [10,15,16]), microbiome research (e.g., [17]), drug design (e.g., [9]), epidemiology (more particularly as a way to understand Koch’s postulates) (e.g., [18]), and so forth.

In this paper, starting with the work of Griffiths et al. [4], which provided a rigorous measure of the notion of specificity *qua* range of influence, I provide some possible information-theoretic measures of invariance. To be clear, these measures should not be regarded as the only possible ones. There are two reasons for this. First, information theory only captures some elements of a relationship between two variables. Garner and McGill [19] illustrate this point well by showing that some relations between information and variance analyses exist. They recommend, however, wherever possible, to use both types of analyses since they both come with advantages and drawbacks. Second, because the notion of invariance is a verbal one, it is prone to different interpretations (within certain bounds). As such I only provide one possible interpretation.

I will treat neither the notion of stability, which has received a substantial treatment for nominal variables in Pocheville et al. [5], nor the notion of one-to-one specificity (again for nominal variables), which is treated in depth in Bourrat [1]. I will also restrict my analysis to nominal variables, but I will set the stage for future work in this area for non-nominal variables.

In Section 2, I present the account of Griffiths et al. [4] and Pocheville et al. [5] of causal specificity *qua* range of influence as a measure of mutual information. I also present an equivalent formulation of mutual information in terms of what is known as “pointwise mutual information”, another information-theoretic measure closely related to mutual information which will be the basis for my discussion of invariance. In Section 3, starting from the notion of pointwise mutual information, I propose two measures that estimate Woodward-invariance. In Section 4, I use a toy example to show how these measures could be deployed. Finally, in Section 5, I show the limits of application of the measures proposed in Section 3 for causal relationships between non-nominal variables.

## 2. Mutual Causal Information and Range of Influence

Both Korb et al. [20] and Griffiths et al. [4] (See also Tononi et al. [21]) independently proposed a measure of range of influence (what Korb et al. call “causal power”) of *C* on *E* as the amount of mutual information transferred from *C* to *E*. I follow here the treatment from Griffiths et al. [4] and Bourrat [1]. Note here that the relationships between information theory and causation have been explored in the foundational work of Collier [22], Collier [23] and Andersen [24].

Mutual information is a measure derived from information theory and in particular Shannon entropy [25]. The entropy of a variable *X* (H(X)) with *n* possible states $x_{1},x_{2},\ldots x_{i},x_{n}$, each with a probability *p* is defined as

(1) $$H\left(X\right)=-\sum_{i=1}^{n}p\left(x_{i}\right)log_{2}p\left(x_{i}\right).$$

Verbally, the entropy of a variable represents the amount of uncertainty about that variable. It is classically measured in bits and can be conceived of as the expected minimum number of questions with a yes/no answer one has to ask to know with certainty the value of the variable. To take a simple example, the outcome of tossing a fair coin has an entropy of only 1 bit because to know with certainty the outcome one only needs to ask a single question such as “Is it heads?” or “Is it tails?”.

The mutual information between two variables, which is derived from Shannon entropy, represents the amount of information gained about one variable upon learning the value of the other. Formally, one way to define the mutual information between *E* and the variable *C* is as

(2) I(E;C)=H(E)−H(E|C),

where I(E;C) represents the mutual information between *E* and *C*, H(E) represents the entropy of *E*, and H(E|C) is the entropy of *E* conditional on *C*, or, in other words, the amount of uncertainty remaining on *E* upon learning the value of *C*.

Griffiths et al. [4], provide a causal version of mutual information between two variables in the causal graph C→E ($I(E;\hat{C})$), which will be the causal model I consider throughout, in which *C* and *E* are related by a joint probability distribution, as follows

(3) $$I\left(E;\hat{C}\right)=H\left(E\right)-H\left(E|\hat{C}\right).$$

Here, ‘ ^ ’ represents the do(.) operator proposed by Pearl [26] which itself represents an ideal intervention performed on a variable. Thus, $\hat{C}$ in Equation (3) represents the variable *C* to which the value has been set by an intervention. Following Bourrat (2019), I will call the measure defined in Equation (3) “mutual causal information.” Griffiths et al. show that this measure corresponds to Woodward’s INF.

As mentioned above, in this paper, I will focus on invariance rather than range of influence. However, Pocheville et al. [5] have proposed, based on the measure of range of influence as mutual causal information, that range of influence (which they call ‘specificity’) and invariance are equivalent (see also [7]). As we shall see, however, the two are different properties of causal relationships. Starting from the notion of mutual causal information will allow me to show why. Furthermore, it turns out that another way of expressing the (causal) mutual information between *C* and *E* is relevant to my formal measures of invariance. One definition of mutual information is the expected value over all possible values of *C* and *E* of what is known as “pointwise mutual information.” For more on this concept see Fano [27] (Chap. 2) in which the term “mutual information” corresponds to the notion of pointwise mutual information used here. We have

(4) $$I\left(E;C\right)=E(pmi\left(e_{j};c_{i}\right)),$$

where $pmi(e_{j};c_{i})$ is the pointwise mutual information between $c_{i}$, which is one of the *N* possible values of *C*, and $e_{j}$, which is one of the *M* possible values of *E*, and E is the expected value. The pointwise mutual information between $c_{i}$ and $e_{j}$ is defined in bits as

(5) $$pmi\left(e_{j};c_{i}\right)=log_{2}\frac{p(c_{i},e_{j})}{p\left(c_{i}\right)p\left(e_{j}\right)}=log_{2}\frac{p(c_{i}|e_{j})}{p(c_{i})}=log_{2}\frac{p(e_{j}|c_{i})}{p(e_{j})},$$

where $p(c_{i})$, $p(e_{j})$, $p(c_{i},e_{j})$, $\frac{p(c_{i}|e_{j})}{p(c_{i})}$ and $p(e_{j}|c_{i})$ are the respective probabilities of events $c_{i}$, $e_{j}$, $c_{i}$ and $e_{j}$, $c_{i}$ knowing $e_{j}$, and $e_{j}$ knowing $c_{i}$.

Using the do(.) operator, for any probability distribution of interventions on *C*. As pointed out by an anonymous reviewer, it is important to note that the standard do-calculus of Pearl does not exactly use distributions over interventions even if it is often presented as such. To properly define interventions from the do-calculus perspective, one must explicitly define intervention variables in the sense given by Pearl [28]. The causal version of Equation (4) reads

(6) $$I\left(E;\hat{C}\right)=E\left(pmi(e_{j};\hat{c_{i}})\right)=E\left(log_{2}\frac{p(e_{j}|\hat{c_{i}})}{p(e_{j})}\right),$$

where $pmi(e_{j};\hat{c_{i}})$ is the pointwise mutual causal information from one value of *C* ($c_{i}$) fixed by intervention to one value of *E* ($e_{j}$).

With this rudimentary introduction to the notion of mutual information, I turn in the next section to the core of this paper, namely, the notion of invariance, and provide formal information-theoretic measures of it.

## 3. Measuring Invariance

In this section, I propose that one way to approach the Woodward-invariance of a causal relationship between *C* and *E* in the causal graph C→E is to start from the causal measure of pointwise mutual information between the different values of *C* and *E* presented in Equation (6). Woodward-invariance, from this perspective, amounts to calculating the mean of two variances or by applying calculating the mean of two standard deviations. Using a standard deviation instead of a variance has the advantage of giving a result in bits, while the result with a variance would be in *squared-bits*. In this paper, I will present the case with standard deviations for this reason.

The first part of the measure of invariance is the standard deviation of the pointwise mutual causal information from *C* to *E* once *C* has been fixed to a particular value by intervention, or in other words, conditioning on $\hat{C}$. The second is the standard deviation of the same pointwise mutual causal information conditioning this time on *E*.

**Figure 1:**
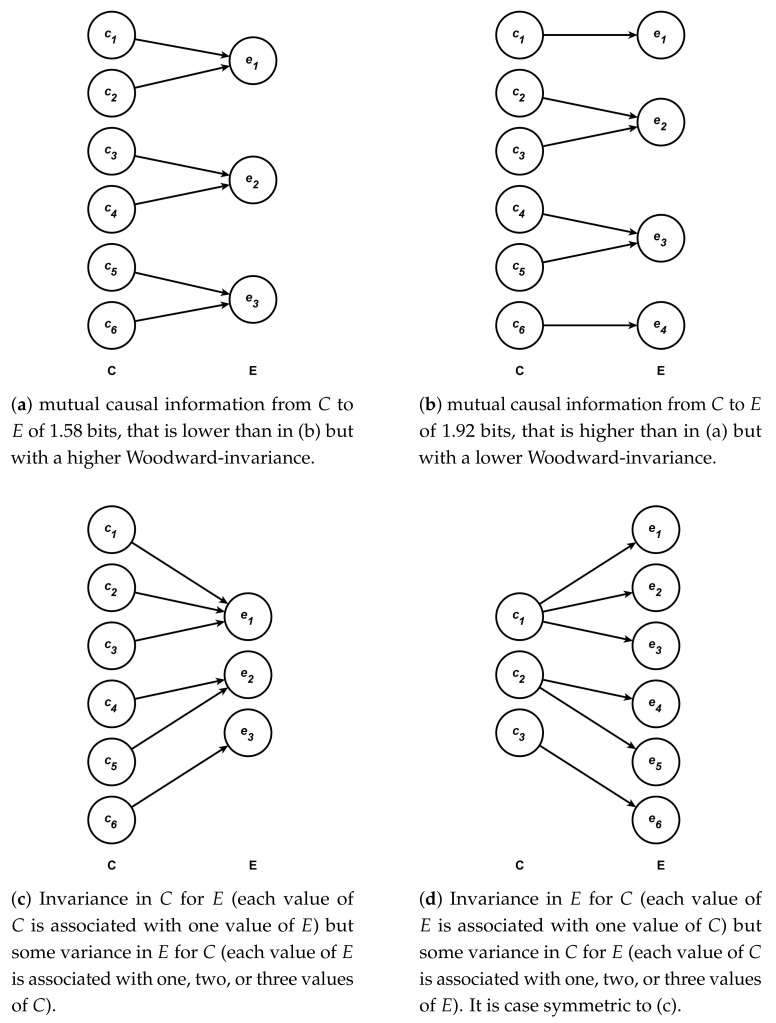
Causal diagrams between *C* and *E* with different degrees of invariance. In each diagram, each value of *C* (e.g., $c_{1}$, $c_{2}$) has the same probability. If there are four possible values of *C*, then each value has a probability of $\frac{1}{4}$. Furthermore, when more than one arrow leaves from a particular value of *C*, each arrow has the same probability conditioned on the value of *C* it leaves from. For instance two arrows leaving a particular value of *C* implies a conditional probability of $\frac{1}{2}$ for each arrow. In a context where there are six possible values of *C*, the overall probability of an arrow leaving a value of *C* from which overall two arrows are leaving is thus $\frac{1}{2}\times \frac{1}{6}=\frac{1}{12}$.

Before giving formal definitions of these two standard deviations, I will provide, with the help of the diagrams presented in Figure 1, an intuitive understanding of what these standard deviations measure. This type of diagram is the same as those used in Griffiths et al. [4], Pocheville et al. [5] and Bourrat [1]. Each of the four diagrams represent the mapping between different values of the variable *C* which has been intervened upon and *E*. Following Bourrat [1], I assume equiprobability for the values of *C*, which is one way to make sense of the notion of possible causation. For a discussion of other probability distributions and how they relate to different senses of causation see Griffiths et al. [4].Each arrow leaving one value of *C* represents the probability of this value causing the value of *E* it points to, conditional on this value of *C* (for more details see the caption of Figure 1). If only one arrow leaves a given value of C, the probability is 1 and the effect it leads to is deterministic. If more than one arrow leaves a value of *C*, then we suppose that each arrow has the same probability conditioning on this value of *C*. The effect *E*, in such a case, is indeterministic because one value of *C* does not always cause one value of *E*. When, in contrast, multiple values of *C* point to the same value of *E*, the effect is multiple realized.

Following the conventions of the diagrams in Figure 1, the first standard deviation corresponds to a measure of whether, across the whole range of possible values of *C* which have been intervened upon, there is variation in the number of arrows leaving one particular value of *C*, assuming each value of *C* has the same probability. The second standard deviation amounts to measuring whether there are differences in both the number and weight of arrows heading toward a particular value of *E* across the whole range of possible values of *E*, assuming each value of *E* has the same probability. Note that the overall weight of each arrow is important here, because one arrow heading towards one value of *E* could represent, overall, a different probability than another arrow pointing toward the same or a different value of *E*.

Using the diagrams in Figure 1, we can make sense of the notion of invariance by looking at the diagram Figure 1a,b. We can see that the causal relationship between *C* and *E* is more invariant in Figure 1a than in Figure 1b. This is because starting *from any value of* *C* in Figure 1a one out of five possible interventions leads to no change in *E* and four out of five leads to a change in *E*. If we now move to Figure 1b, *for some values* of *C*, namely $c_{1}$ and $c_{6}$, five possible interventions out of five lead to changes in the value of *E* and *for the other values* four out of five possible interventions lead to a change and one out of five will lead to no change. Thus, because there is more variation in the type of outcome (change/no change), intervening on *C* leads to in Figure 1b than in Figure 1a, and the relationship between *C* and *E* in Figure 1a is more invariant than it is in Figure 1b. Furthermore, the mutual causal information from *C* to *E* in Figure 1b (1.92 bits) surpasses that of Figure 1a (1.58 bits), which clearly shows that range of influence and invariance ought to be considered as distinct properties contrary to what has been claimed in the literature (e.g., [5,7]). This is so because a higher range of influence does not equate to a higher level of invariance, as I define it here.

Importantly, it should be noted that if the two standard deviations measuring invariance are low, this implies on the one hand that the variation on the mapping between one given value of *C* and the different possible values of *E* is very similar across all values of *C*, and on the other hand that the mapping between one given value of *E* and the different possible values of *C* is also very similar across all values of *E*. Using only one of the two standard deviations is insufficient to assess the overall invariance of the relationship. This is so because there might be little or no variation in the mapping between one given value of *C* and the different possible values of *E* with which it is associated, yet substantial variation between one given value of *E* and the different possible values of *C* with which it is associated, and vice versa. Using the diagrams in Figure 1, this situation is one in which many values of *C* are mapped in the same way to *E* (each value of *C* might, for instance, be associated with one single *E* as in Figure 1c), while each value of *E* is mapped in a different way to *C* (each values of *E* might, as one possibility, be associated with a different number of values of *C* as in the same diagram), and vice versa as in Figure 1d. In such cases, the reduction of uncertainty in the value of *C* (*E*) upon learning the value of *E* (*C*), once an intervention is carried out on *C*, will vary for different values of *E* (*C*). Furthermore, each standard deviation should be given the same weight in the measure of invariance as there is no principled reason to favor either the relationship of a causal value with its effect(s) or the causal relationship of an effect value with its cause(s).

I now present the two standard deviations formally. The details of the derivations for these formulas are presented in the Appendix A. We get the standard deviation of the expected pointwise mutual information between one known value of *C* ($c_{i}$) fixed by intervention and one value of *E* across the whole range of *M* possible values of *E* ($\sigma \left[E_{c_{i}}\{pmi\left(e_{j};\hat{c_{i}}\right)\}\right]$) as

(7) $$\begin{array}{cc} \; & \sigma \left[E_{c_{i}}\{pmi\left(e_{j};\hat{c_{i}}\right)\}\right]=\\ & \sqrt{\sum_{i=1}^{N}p\left(\hat{c_{i}}\right){\left(\left[\sum_{j=1}^{M}p\left(e_{j}|\hat{c_{i}}\right)log_{2}\frac{p(e_{j}|\hat{c_{i}})}{p(e_{j})}\right]-MI\left(E;\hat{C}\right)\right)}^{2}}. \end{array}$$

If $\sigma \left[E_{c_{i}}\{pmi\left(e_{j};\hat{c_{i}}\right)\}\right]=0$, this implies that all values of *C* have the same type of association with *E*, that is, for each *C* value, the same probability on average, for each one of these effects. In that sense the relationship between *C* and *E* is invariant. If, instead $\sigma \left[E_{c_{i}}\{pmi\left(e_{j};\hat{c_{i}}\right)\}\right]>0$, this implies that not all of the values of *C* have the same type of association with *E*. The higher the value of the square root, the lower the invariance of the mapping between a given value of *C* and the values of *E*.

For the second standard deviation, expressly, the standard deviation of the expected pointwise mutual information between one known value of *E* ($e_{j}$) and one value of *C* fixed by intervention across the whole range of *N* possible values of *C* ($\sigma \left[E_{e_{j}}\{pmi\left(e_{j};\hat{c_{i}}\right)\}\right]$), we get

(8) $$\begin{array}{cc} \; & \sigma \left[E_{e_{j}}\{pmi\left(e_{j};\hat{c_{i}}\right)\}\right]=\\ & \sqrt{\sum_{j=1}^{M}p\left(e_{j}\right){\left(\left[\sum_{i=1}^{N}p\left(\hat{c_{i}}|e_{j}\right)log_{2}\frac{p(\hat{c_{i}}|e_{j})}{p(\hat{c_{i}})}\right]-MI\left(E;\hat{C}\right)\right)}^{2}}. \end{array}$$

Using the same prior reasoning, if $\sigma \left[E_{e_{j}}\{pmi\left(e_{j};\hat{c_{i}}\right)\}\right]=0$, this implies that all the values of *E* have the same type of association with *C*. In that second sense the relationship between *C* and *E* is invariant. If now $\sigma \left[E_{e_{j}}\{pmi\left(e_{j};\hat{c_{i}}\right)\}\right]>0$, this implies that not all of the values of *E* have the same type of association with *C*. As before, the greater the value of this standard deviation, the less invariant the relationship becomes.

With these two formulas in place, we can now characterize Woodward-invariance in information-theoretic terms as the mean between the standard deviations of Equation (7) and that of Equation (8), so that

(9) $$V\left(\hat{C};E\right)=\frac{\sigma \left[E_{c_{i}}\{pmi\left(e_{j};\hat{c_{i}}\right)\}\right]+\sigma \left[E_{e_{j}}\{pmi\left(e_{j};\hat{c_{i}}\right)\}\right]}{2},$$

where $V(\hat{C};I)$ is an information-theoretic measure of Woodward-(in)variance from *C* to *E*. We can deduce from this measure if interpreted as Woodward-invariance, that is, if the relationship between *C* and *E* is bijective (maximally one-to-one specific), then it is maximally invariant when $V(\hat{C};I)=0$ bits (the inverse proposition is; however, not true). I should thus be clear that strictly speaking, the measure I propose is not a measure of *in*variance, but rather a measure of variation, where a variation equal to 0 signifies a maximally invariant relationship between *C* and *E*.

Note that the measure presented in Equation (9) is only one possible information-theoretic measure of Woodward-invariance. An alternative measure involves the use of a *normalized* version of pointwise mutual information. The normalized pointwise mutual information (npmi) between one value *i* of *C* fixed by intervention and one value *j* of *E* is defined as

(10) $$npmi\left(e_{j};\hat{c_{i}}\right)=\frac{pmi(e_{j};\hat{c_{i}})}{h(e_{j},\hat{c_{i}})}=\frac{log_{2}\frac{p(e_{j}|\hat{c_{i}})}{p(e_{j})}}{-log_{2}\left(p\left(e_{j},\hat{c_{i}}\right)\right)},$$

where $h\left(e_{j},\hat{c_{i}}\right)=-log_{2}\left(p\left(e_{j},\hat{c_{i}}\right)\right)$ is the joint self-information of $e_{j}$ and $\hat{c_{i}}$. Equation (10) is simply the pointwise mutual information between these two values divided by the joint self-information of the two events.

Using the normalised pointwise mutual information, we can derive two equations similar to Equations (7) and (8), as

(11) $$\begin{array}{cc} \; & \sigma \left[E_{c_{i}}\{npmi\left(e_{j};\hat{c_{i}}\right)\}\right]=\\ \; & \sqrt{\sum_{i=1}^{N}p\left(\hat{c_{i}}\right){\left(\left[\sum_{j=1}^{M}1-\frac{log_{2}\left(p\left(e_{j}\right)\right)}{log_{2}\left(p\left(e_{j}|\hat{c_{i}}\right)\right)}\right]-\frac{MI(E;\hat{C})}{H(E,\hat{C})}\right)}^{2}}, \end{array}$$

and

(12) $$\begin{array}{cc} \; & \sigma \left[E_{e_{j}}\{pmi\left(e_{j};\hat{c_{i}}\right)\}\right]=\\ \; & \sqrt{\sum_{j=1}^{M}p\left(e_{j}\right){\left(\left[\sum_{i=1}^{N}1-\frac{log_{2}\left(p\left(\hat{c_{i}}\right)\right)}{log_{2}\left(p\left(\hat{c_{i}}|e_{j}\right)\right)}\right]-\frac{MI(E;\hat{C})}{H(E,\hat{C})}\right)}^{2}}, \end{array}$$

respectively. This leads to the following normalized version of Equation (9) as

(13) $$V_{n}\left(\hat{C};E\right)=\frac{\sigma \left[E_{c_{i}}\{npmi\left(e_{j};\hat{c_{i}}\right)\}\right]+\sigma \left[E_{e_{j}}\{npmi\left(e_{j};\hat{c_{i}}\right)\}\right]}{2}.$$

The details of the derivations are presented in Appendix B.

The main difference between the normalized version of Woodward’s invariance measure and the version presented in Equation (9) is that the normalized version discounts variation resulting from variations in the self-information of $e_{j}$ and $\hat{c_{i}}$, conditioned on $\hat{C}$ and variation in the self-information of $e_{j}$ and $\hat{c_{i}}$, conditioned on *E*. If such differences exist, dividing the conditional pointwise mutual information of a pair by its corresponding conditional joint self-information will permit us to discount the measure of invariance due to the variation(s) in joint self-information. Thus, provided the causal relationship, in and of itself, has values of joint self-information which are unequal for each pair of *C* and *E* conditioned on *C*, and/or unequal for each pair of *C* and *E* conditioned on *E*, the normalized measure eliminates any variance that does not originate from the relationship itself, but from the fact it is measured in a particular population in which there is some variation in at least one of the two conditional joint probability distributions of pairs of values (measured by the self-information of these two values).

However, it would be a mistake to claim that the normalized version of the measure is superior to the non-normalized one, because it might be a property of the causal relationship that conditional joint self-information differs for one pair of values in contrast with the others. Applying the normalized version of the measure in those cases would produce a lower estimate, implying a higher Woodward-invariance for the relationship than what it actually is. The problem of characterizing causal relationships when the distribution of variables is not uniform is not restricted to measures of invariance.

Another difference between the two measures worth mentioning is that the normalized version does not give an estimate in bits, but is a ratio. Using a ratio instead of an absolute measure might be appropriate for some purposes but not others.

Overall, the two measures have distinct strengths and weaknesses. In light of the difference between the two measures, a complete characterization of Woodward’s invariance with a single information-theoretic measure looks to be impossible. As we will see in the last section, further complications arise when we introduce non-nominal variables. Nevertheless, the two measures are useful in characterizing desirable properties of causal relationships that can be related to invariance. In the next section, I provide an example that demonstrates this.

## 4. Example

Suppose one wants to characterize the invariance of the relationship between certain alleles of a gene G1 and hair curliness in humans. A number of genes, such as EDAR, FGFR2 and TCHH have been associated with hair curliness in different populations (e.g., [29,30,31]). Although recent studies classify human hair in eight types [32,33] distinguished by several parameters of the curliness of the hair, the traditional separation of hair curliness in the following four types remains: ‘straight’, ‘wavy’, ‘curly’, and ‘kinky’.

Suppose that one establishes that, in a given population, six alleles of G1 are associated with three of the four types of hair (straight, wavy, and curly). I assume here that the association between gene variants and hair phenotype is causal. Suppose now that the pattern of association follows exactly the causal diagram (a) presented in Figure 1, so that each allele $g1_{a}$, *…*, $g1_{f}$ replaces $c_{1}$, *…*, $c_{6}$, respectively, and that each phenotype straight, wavy, and curly replaces $e_{1}$, $e_{2}$, and $e_{3}$ with the same probability, respectively. This is, of course, an unrealistic example, but it serves to illustrate how the measure of invariance presented in the previous section could be employed in a real case. Given the causal diagram (a), we can thus establish that the range of influence of *G* on hair curliness is 1.58 bits using the formulas presented in Equation (3) or Equation (6). Using now the measure of Woodward’s (in)variance proposed in Equation (9), we find, 0 bit. This is because the pointwise mutual causal information between one allele fixed by intervention and hair phenotype, knowing the allele, is always 1.58 bits of information across the six alleles. Similarly, the pointwise mutual causal information between one hair phenotype and one allele fixed by intervention, knowing the hair phenotype is always 1.58 bits across the three hair phenotypes. Thus, overall, for a given allele (a given state of hair phenotype), there is no variation in the number of states and weight for each state of hair phenotype (alleles) it is associated with. An allele is always associated with a single state of hair phenotype, while a state of hair phenotype is always associated with two different alleles. Note here that because the two types of conditional joint self-information are the same for all pairs, the normalized version of the measure would yield the same answer (modulo the fact it would be a ratio and not an absolute measure in bits).

Suppose now that we identify a second gene G2, homologous to G1, which also has six different alleles associated with the phenotype hair type, but this time according to the causal diagram (b) presented in Figure 1 with $e_{4}$ corresponding to the hair phenotype kinky, so that each allele ($g2_{a}$, *…*, $g2_{f}$) replaces ($c_{1}$, *…*, $c_{6}$) respectively, and that each phenotype (straight, wavy, curly, and kinky) replaces ($e_{1}$, $e_{2}$, $e_{3}$, and $e_{4}$) respectively with the same probability. If we calculate now the range of influence of G2 on hair curliness, we obtain 1.92 bits using the formulas presented in Equation (3) or Equation (6). A measure of Woodward’s (in)variance proposed in Equation (9), gives in this case 0.91 bits of information, which implies a lower invariance than between G1 and hair phenotype. This can be explained by the fact that although each allele is associated with a single hair phenotype, each hair phenotype is not associated with the same number of alleles across the whole state space of hair type. The types straight and kinky are associated with a single allele, while the types wavy and curly are associated with two alleles, respectively.

With this measure of invariance in hand, we could conclude that although G2 has a larger range of influence than G1 on hair curliness, the causal relationship between G2 and hair phenotype is nevertheless less invariant than it is between G1 and hair phenotype. Of particular importance, we could see that some alleles are associated with a single phenotype while others are associated with two, an observation that may suggest that the causal relationship between G2 and hair type involves two underlying mechanisms involving the allele and the phenotypic outcome. One mechanism would operate with some alleles ($g2_{a}$ and $g2_{f}$) while another would operate with other alleles. This would represent a contrast with the relationship between G1 and hair phenotype, in which the most parsimonious interpretation of the measure is that a single underlying mechanism relates G1 and hair phenotype across the whole range of alleles. Of course, such an interpretation relies on a parsimony assumption and it may well be the case that the number of mechanisms involved for G1 is higher than for G2. However, assuming that the two genes are functionally similar (an assumption that could be justified by the fact that the two genes are homologous), in the absence of more information, there would be no particular reason to think so.

In the next section, I turn to invariance for causal relationships between non-nominal variables. I will not attempt to characterize formally what the Woodward-invariance for non-nominal variables amounts to, but merely show some important limitations of the information-theoretic approach I have proposed in those cases.

## 5. Invariance and Non-Nominal Variables

I have supposed thus far that the variables involved in the causal relationship between *C* and *E* are nominal variables, that is, variables on which no arithmetic operations (such as addition and multiplication) can be performed. With nominal variables, by definition, each value of the variable is incommensurable with other values of the same variable. This means that a relationship between two nominal variables *C* and *E*, were it known, could not be represented as a function (and consequently can not be represented as the graph of this function), but only as a contingency table (i.e., a crosstab). For this reason, the only way by which the Woodward-invariance of a nominal causal relationship can be estimated is by using the information-theoretic measures provided in Equations (9) and (13).

In contrast to nominal variables, the values of ordinal variables, as their name suggests, can be ordered. However, as with nominal variables, no arithmetic operations can be performed on them. It is nevertheless *possible* to represent (with some loss of information) the relationship between two ordinal variables *C* and *E* as a function by associating the rank of each value of one variable with the mean rank of the other. Because of this property, it is possible to find cases in which the mapping between *C* and *E* values does not vary in terms of point-wise mutual information, but instead varies with respect to other properties of the mapping such as the difference in mean rank between *C* and *E* for each value of *C* (or *E*), its variance, or any other moment of the variable’s distribution.

Yet, information theory does not permit us to assess these differences. In fact, considering the value of a variable, its particular rank and the particular ranks of the values of another variable with which it is associated, is part of the semantic content of information. Shannon’s information theory characterizes information at the syntactic level. It characterizes the relation between the information contained in two or more variables, not the meaning (or semantic content) of this information [34]. For instance, the mapping between two variables and the information-theoretic measure we can subsequently draw is a characterization of information at the syntactic level. Assuming for ease of exposition that the mapping is bijective, the only other property of the relationship that may vary with two ordinal variables is the particular rank of *E* to which each value of *C* is assigned. The rank of a variable’s particular value is part of the semantic content of this variable. Because it is part of its semantic content, any difference in this property between different causal relationships will not be captured by the measure obtained from Equation (9) (or Equation (13)), which stems directly from Shannon’s information theory.

To illustrate this point, suppose for example, that there is a bijective causal relationship between *C* “size of the settlement in which an individual lives” and *E* “reported level of happiness experienced by an individual.” Interpreted causally, a bijective mapping implies that the reported level of happiness is fully determined by the type of settlement in which people live. Here again, the example is implausible, but I employ it to demonstrate potential and make a conceptual point. Assume the domain of *C* to be {hamlet, village, town, city, metroplis} and the codomain of *E* to be {veryhappy, happy, neutral, unhappy, veryunhappy}. The ranking for *C* goes from 1 for the smallest type of settlement (hamlet) to 5 for the largest (metropolis), and the ranking for *E* goes from 1 for the lowest reported level of happiness to 5 for the highest. With five values for *C* and five values for *E*, there are 120 possible bijective mappings between *C* and *E*. For each one of these 120 possible bijective mappings (assuming equiprobability of *C* and *E* values), the measure proposed in Equation (9) will be nil. Yet, despite this, the function of the ranking of *C* leading to a ranking of *E* might either be the same for the entire ranking of *C*, following, for example, the identity or inverse function, or different for some of or all the rankings of *C*.

In the first type of case, the Woodward-invariance of the relationship would be maximal. If the mapping function from the ranking of *C* to the ranking of *E* was, as one option, the identity function, the causal interpretation would be that living in a larger settlement is causing people in that settlement to report that they are happier. However, there is no reason to suppose that the function associating a rank of *C* to a rank of *E* is the same for all ranks of *C*. The mapping might, as another possibility, be the identity function for the ranking sub-domain {2, 3, 4} and the inverse function for the ranking sub-domain {1, 5} or any other combination of functions compatible with a bijective mapping. In this second type of case, there will be some variation in what function maps the ranking of *C* to the ranking *E* across the domain of the ranking of *C*, and for that reason the invariance of the relationship will be lower (just how much lower will depend on the number of functions involved in the relationship).

Moving now to discrete and continuous quantitative variables that have the same properties as ordinal variables and, furthermore, on which arithmetic operations can be performed, the very same limitations to measure Woodward-invariance using Equation (9) or Equation (13) will apply. The only difference between strictly ordinal and quantitative variables is that in the case of quantitative variables the relationship between *C* and *E* can be represented using the values of the two variables directly, rather than their ranks (we have more quantifiable semantic information than in the case of ordinal variables).

Before concluding, it should be noted that I have assumed here that for each value *C* the mapping function between *C* and *E* (or between the ranking on *C* and the ranking on *E* for strictly ordinal variables) is known. Yet, in practice, such functions are never known. This is ultimately linked to the curve-fitting problem which itself is related to the problem of induction (see, [35]). Since such functions are never known, to establish causality, scientists must instead find the best fitting curve, assuming some constraints (such as parsimony) for a set of data points. Thus, for any set of data points, in practice, one and only one function is assumed to explain the mapping between *C* and *E* for all values of *C*. Since Woodward-invariance is concerned precisely with the number of functions that apply for a given causal relationship, using any curve fitting technique will imply that the invariance of the relationship is maximal. Characterizing precisely the invariance of a relationship with dimensions not captured by the information-theoretic measures proposed in Equations (9) and (13) (when the variables involved are ordinal), as well as pragmatically, will thus require finding some properties of fitting functions that are commensurable (presumably under some particular assumptions) with invariance.

By proposing the example of the causal relationship between the ordinal variables “size of the settlement in which an individual lives” and “level of happiness experienced by an individual” in which the identity function applies for some values and the function inverse for some other values, one can catch a glimpse of what such a commensurable property between the fitting of a single function and Woodward-invariance is. If we were to fit one single function between the rank of *C* and the rank of *E* in this case, the function would be a U-shaped one (i.e., nonlinear). This would suggest that nonlinearity could be an indicator of a low Woodward-invariance. That said, nonlinearity in and of itself cannot serve as a basis for characterizing invariance since many relationships are nonlinear (and might be highly invariant). A more promising avenue for a reliable indicator of Woodward-invariance would be a unifying mechanism relating the two variables. The lack of a known unifying mechanism relating two variables associated with a nonlinear (fitted) relationship would in that case be an indicator of a low Woodward-invariance, since the relationship between the two variables would depend on a number of distinct mechanisms, each of which would perform different activities and produce different outcomes for different values of *C*.

Far from having characterized precisely what one can mean by causal invariance for ordinal and quantitative variables, I have at least paved the way for future work on the topic.

## 6. Conclusions

The aim of this paper was to provide precise measures that are faithful to the notion of Woodward-invariance. I first introduced the notion of mutual causal information proposed by Griffiths et al. [4] and presented an alternative definition of it in terms of point-wise mutual information. I then proposed, starting from pointwise mutual information, two information-theoretic measures of Woodward-invariance, one of which is a normalized version of the other. From there, I presented a simple example to illustrate how these measures could be useful. Finally, I argued that these measures can only fully characterize one aspect of what can be meant by ‘invariance’ following Woodward’s treatment. In particular, I demonstrated that, for non-nominal variables, the characterization of the mapping between two variables necessarily involves considerations about the ranks of the variables for ordinal variables and the values for quantitative variables, which cannot be captured by the formalism of Shannon’s information theory.

Given that there are some points of similarity between the notions of causal specificity, invariance, and stability, my considerations for non-nominal variables will apply to these notions as well.

## Data Availability

Not applicable.

## Appendix A

Using the do(.) operator, we start with the pointwise mutual causal information between one value *i* of *C* fixed by intervention and one value *j* of *E* which may be defined as

(A1) $$pmi\left(e_{j};\hat{c_{i}}\right)=log_{2}\frac{p(e_{j}|\hat{c_{i}})}{p(e_{j})}.$$

We can then define the expected pointwise mutual information between one known value of *C* ($c_{i}$) fixed by intervention and one value of *E* across the whole range of *M* possible values of *E*. This leads to

(A2) $$E_{c_{i}}\left\{pmi\left(e_{j};\hat{c_{i}}\right)\right\}=\sum_{j=1}^{M}p\left(e_{j}|\hat{c_{i}}\right)log_{2}\frac{p(e_{j}|\hat{c_{i}})}{p(e_{j})}.$$

We can then calculate the variance of $E_{c_{i}}\{pmi\left(e_{j};\hat{c_{i}}\right)\}$($Var\left[E_{c_{i}}\{pmi\left(e_{j};\hat{c_{i}}\right)\}\right]$). Since the expected value of $E_{c_{i}}\{pmi\left(e_{j};\hat{c_{i}}\right)\}$ over the *N* possible values of *C* is simply the mutual causal information from *C* to *E*, we have

(A3) $$\begin{array}{cc} \; & \mathrm{Var}\left[E_{c_{i}}\{pmi\left(e_{j};\hat{c_{i}}\right)\}\right]=\\ \; & \sum_{i=1}^{N}p\left(\hat{c_{i}}\right){\left(\left[\sum_{j=1}^{M}p\left(e_{j}|\hat{c_{i}}\right)log_{2}\frac{p(e_{j}|\hat{c_{i}})}{p(e_{j})}\right]-MI\left(E;\hat{C}\right)\right)}^{2}. \end{array}$$

Applying the square-root function to Equation (A3) results in the standard deviation of $E_{c_{i}}\{pmi\left(e_{j};\hat{c_{i}}\right)\}$ ($\sigma \left[E_{c_{i}}\{pmi\left(e_{j};\hat{c_{i}}\right)\}\right]$) as Equation (7).

To calculate the second standard deviation, we start from Equation (A1) and then define the expected pointwise mutual information between one known value of *E* ($e_{j}$) and one value of *C* fixed by intervention across the whole range of *N* possible values of *C* ($E_{e_{j}}\{pmi\left(e_{j};\hat{c_{i}}\right)\}$), which is

(A4) $$E_{e_{j}}\left\{pmi\left(e_{j};\hat{c_{i}}\right)\right\}=\sum_{j=1}^{M}p\left(e_{j}\right)\left[\sum_{i=1}^{N}p\left(\hat{c_{i}}|e_{j}\right)log_{2}\frac{p(\hat{c_{i}}|e_{j})}{p(\hat{c_{i}})}\right].$$

From there we can calculate the variance of $E_{e_{j}}\{pmi\left(e_{j};\hat{c_{i}}\right)\}$ ($\mathrm{Var}\left[E_{e_{j}}\{pmi\left(e_{j};\hat{c_{i}}\right)\}\right]$). The expected value of $E_{e_{j}}\{pmi\left(e_{j};\hat{c_{i}}\right)\}$ over the *M* possible values of *E* is simply the mutual causal information from *C* to *E*. We thus have

(A5) $$\begin{array}{cc} \; & \mathrm{Var}\left[E_{e_{j}}\{pmi\left(e_{j};\hat{c_{i}}\right)\}\right]=\\ & \sum_{j=1}^{M}p\left(e_{j}\right){\left(\left[\sum_{i=1}^{N}p\left(\hat{c_{i}}|e_{j}\right)log_{2}\frac{p(\hat{c_{i}}|e_{j})}{p(\hat{c_{i}})}\right]-MI\left(E;\hat{C}\right)\right)}^{2}. \end{array}$$

Applying the square root function to Equation (A5) we get the standard deviation of $E_{e_{j}}\{pmi\left(e_{j};\hat{c_{i}}\right)\}$ ($\sigma \left[E_{e_{j}}\{pmi\left(e_{j};\hat{c_{i}}\right)\}\right]$) as Equation (8).

Combining Equations (7) and (8), we get Equation (9).

## Appendix B

We start from the normalized pointwise mutual causal information from $c_{i}$ to $e_{j}$ in Equation (10).

Using a reasoning similar to the one used with the non-normalized version of Woodward-invariance in Appendix A, *mutatis mutandis*, we first calculate the normalized version of the standard deviations of Equations (7) and (8). In the case of Equation (7), this leads to

(A6) $$\begin{array}{cc} \; & \sigma \left[E_{c_{i}}\{npmi\left(e_{j};\hat{c_{i}}\right)\}\right]=\\ \; & \sqrt{\sum_{i=1}^{N}p\left(\hat{c_{i}}\right){\left(\left[\sum_{j=1}^{M}\frac{p\left(e_{j}|\hat{c_{i}}\right)log_{2}\frac{p(e_{j}|\hat{c_{i}})}{p(e_{j})}}{p\left(e_{j},\hat{c_{i}}|\hat{c_{i}}\right)log_{2}\left(p\left(e_{j},\hat{c_{i}}|\hat{c_{i}}\right)\right)}\right]-\frac{MI(E;\hat{C})}{H(E,\hat{C})}\right)}^{2}}, \end{array}$$

where, $p\left(e_{j},\hat{c_{i}}|\hat{c_{i}}\right)log_{2}\left(p\left(e_{j},\hat{c_{i}}|\hat{c_{i}}\right)\right)$ is the joint-self entropy of the pair $\hat{c_{i}},e_{j}$, conditioned on $\hat{c_{i}}$, $H(E,\hat{C})$ is the joint entropy between *C* to which the value has been set by intervention and *E*, that is the expected joint self-information for all possible pairs of $\hat{C}$ and *E*. The joint entropy of a pair of variables is simply the Shannon entropy of two variables instead of one. Since $p\left(e_{j},\hat{c_{i}}|\hat{c_{i}}\right)=p\left(e_{j}|\hat{c_{i}}\right)$ this simplifies into Equation (11).

Following the same steps, we reach the normalized version of Equation (8) as Equation (12).

These two equations, once combined, lead to the normalized version of Equation (9) as Equation (13).
